# We’ve Only Just Begun – Insights from a 25-Year Journey to Accelerate Health Care Transformation Through Delivery System Research

**DOI:** 10.5334/egems.310

**Published:** 2019-04-24

**Authors:** Sarah M. Greene, Paul Wallace, Andrew F. Nelson

**Affiliations:** 1 Health Care Systems Research Network, US; 2 AcademyHealth, US; 3 HealthPartners Institute, US

**Keywords:** Research, health, health care data, learning health system, team science, collaboration

## Abstract

Even though it is well known that quality, safety, and patient-centeredness of health care can be improved, leveraging the organizational apparatus of a care delivery environment to render improvement in a consistent and comprehensive manner has proven difficult. The Health Care Systems Research Network (HCSRN), which began as the HMO Research Network, emerged from a desire to improve health and study problems in health care in a systematic and collaborative way, spurring the delivery of true evidence-informed medicine. The HCSRN has honed network-wide data resources, a collaborative culture, and shared infrastructure, enabling multicenter health care research that is often more difficult for researchers working in less integrated settings and across organizational boundaries. The HCSRN’s 25-year track record confers both an opportunity and obligation to share what we have learned through our research. Considering the quarter-century since the HCSRN was established, we describe three evolving areas—health data, new health care models, and diversified research teams that must be thoughtfully harnessed to realize a transformed health care ecosystem that generates and learns with research.

## Introduction

Optimizing evidence-informed care in a fragmented health care system is an enduring challenge, as landmark reports from the National Academy of Medicine recognized in “To Err Is Human” and “Crossing the Quality Chasm” [1,2]. Even though it is well known that quality, safety, and patient-centeredness can be improved, leveraging the organizational apparatus in a health care environment to render improvement in a consistent and comprehensive manner has proven difficult. Hence, provision of health care is often unsystematic, in that the best available evidence about effective care is not routinely applied [3,4].

The HCSRN, which began as the HMO Research Network, emerged from a desire to improve health and study problems in health care in a systematic and collaborative way [5], spurring the delivery of true evidence-informed medicine. Inspired by the application of systems thinking to create learning organizations [6], the HCSRN has honed network-wide data resources, collaborative culture, and shared infrastructure. This enables large-scale health care research that is often more difficult for researchers working in less integrated settings and across organizational boundaries. The HCSRN’s 25-year portfolio of clinical, epidemiological and health services research not only reflects hard work and privilege, it confers both an opportunity and obligation to share what we have learned through our work. Moreover, as the HCSRN has accrued research results, it has developed a technical and relational infrastructure that enable us to *scale and apply* what we learn to our member health systems and beyond.

Hence, this *eGEMS* journal supplement is more than a compendium that has roots in a multisite network. The methods and insights from the HCSRN and its partners demonstrate the value of shared infrastructure and shared culture. Commonalities in data capabilities and the shared ideal that research is a public good spurred HCSRN’s desire and ability to collaborate. Considering the quarter-century since the HCSRN was established, we describe three areas that have evolved, and must continue to evolve in order, to realize a transformed health care ecosystem that generates and learns from research.

## Three evolving aspects of health system research

**Health data functions as both a cornerstone and a commodity.** The ubiquity of health data is both an asset and a potential liability. Data are proliferating through sources we did not fully imagine even ten years ago, thanks to wearable devices, social media, the “Internet of Things,” and of course, electronic health records (EHRs). This has spawned a commensurate interest in using data to predict events and outcomes—cottage industries related to algorithms, analytics, and visualization have emerged. To successfully improve health and health care in our big data era, we need to focus not only on volume, velocity, and variety—the so-called 3V’s of big data [7], but also veracity, validity, and value. Veracity asks us to consider whether the data are accurate and reliable. Validity helps us understand whether the data has the power and representativeness to address a given question. And importantly, the data must yield an answer that has some *value* to stakeholders. Studies of post-marketing surveillance exemplify this—the larger the population, the more precisely incident events can be identified. Embedded in each of these attributes is the need to understand data provenance (i.e., source of the data) and context (i.e., under what circumstances were the data collected?). The HCSRN’s Virtual Data Warehouse has long functioned as a federated “big data” repository for our participating systems, and since it is a primary research resource for many of our studies, the attention to metadata and curation is paramount [8,9]. As well, several articles in this special issue illustrate the importance of deep familiarity with the source data (see Appendix 1 below for the full list of special issue papers by topic area).

**New health care and coverage models have significant implications for data-driven health services research.** The prevailing mode of care delivery in ambulatory clinics and hospitals is quickly giving way to health care that is dispersed—virtual care, retail clinics, the hospital at home model, and even health advice that leverages artificial intelligence, are four relatively recent developments poised to alter the health care landscape with lasting impact. Moreover, payment models and health care coverage are shifting from paying for volume of care (leading to a propensity for overuse), to paying for value and managing the health of populations through accountable care entities. This shift from traditional health system delivery arrangements offers new opportunities to conduct research on optimal structure and financing of health care that addresses *individual* circumstances (e.g., sociodemographics, economic disparities, social influences, health status, patient preferences), and *organizational* features (where, how, and by whom care is delivered). As well, this shift brings new challenges for data availability and applicability of findings in new and different care settings. In the HCSRN, comparable data (e.g., on cancer screening, medication orders, or an incident diagnosis) are available with relative consistency, facilitating examination of health system-initiated changes at the patient, clinician, or organizational level [10]. Along with the publications in this issue on embedding and measuring specific evidence-based interventions, clinical decision support, and complex care management, the HCSRN’s current portfolio includes studies of telemedicine, the use of community-based navigators to support disease management, and impact of benefit designs on medication adherence, all of which offer lessons that can be applied in diverse settings.

**Diversified research teams will accelerate our ability to translate research into better health and health care:** Two concurrent influences have ushered in new thinking related to the composition of scientific research teams. The first, team science, has been promulgated by the National Cancer Institute as an intentional approach to creating “coordinated teams of investigators with diverse skills and knowledge…for studies of complex problems with multiple causes.” [11]. The second is the welcome emphasis on patient- and stakeholder-engaged research, accelerated by creation of the Patient-Centered Outcomes Research Institute (PCORI). By creating multidisciplinary teams and involving end-users of evidence (patients, clinicians, and other decision-makers), health care research can increase both its rigor and its relevance. The HCSRN has decades-long collaborations where multidisciplinary teams might include health services researchers, medical anthropologists, data scientists, and behavioral scientists. In the case of our Mental Health Research Network, a patient/stakeholder advisory board was added recently. Several HCSRN sites have also established formal programs related to the learning health system [12,13], entailing close collaboration with system leaders and frontline clinicians. Many of these sites will be training a new generation of delivery system scientists who develop specific core competencies related to the learning health system [14].

**What do these three forces mean for the HCSRN and the research community?** This is an exhilarating time for health research. Technology and data are enabling momentous discoveries with the potential to change medical care as we know it. Ever larger research datasets help us undertake observational studies with greater precision [15]. Pragmatic clinical trials are a newly potent method for understanding how treatments work under real-world conditions. And yet, we have not yet solved for rampant health inequities, clinician burnout, or high health care costs. Research—a long-standing public good—can help abate this asymmetry. To achieve this, we must continue to forge new relationships with clinical leaders and take down the silos that have slowed the pace of translation. This could mean shifting from a supply-side mindset (conducting research and hoping clinician uptake results) to a demand-driven mindset (undertaking research that is congruent with the urgent needs of health system leaders). The Veterans’ Administration Quality Enhancement and Research Initiative (VA QUERI) has pioneered just such a shift, and can serve as an exemplar of this paradigm, purposely linking research activities to clinical care and facilitate adoption of evidence-based care improvements [16,17,18].

In our experience, the HCSRN has become a useful population laboratory to test interventions in real-world systems—taking action based on an identified care gap or potential health system improvement. And while our member systems are sometimes held up as rarefied settings or “unicorns,” where those outside of the system wonder if there are unique facets of [Kaiser Permanente/Geisinger/HealthPartners/etc.] that are not necessarily generalizable to other settings, we have also made progress moving research into practice, which can help inform the research community at large. Health care consultants are often enlisted when health system leaders want focused problem-solving on cost, quality or operational challenges, but what would it take for these leaders to turn to researchers first? We suggest the following steps to encourage closer integration of research and practice, and invigorate collaboration with all stakeholders–researchers, system leaders and clinicians, patients, and communities:

1. Researchers must be conversant in the **evidence base *and* the business case** for their areas of expertise.
2. Researchers must be able to articulate not only the statistical significance, but also the **potential clinical, operational, and practical significance** of our results.
3. Researchers must recognize that what we have traditionally valued—publication in high-impact journals and steady funding—may not be as consequential to health care decision-makers or communities, especially relative to care improvement goals. Yet we must be able **convey the value proposition for research** in ways that resonate with system leaders or patient groups.

**Figure 1:**
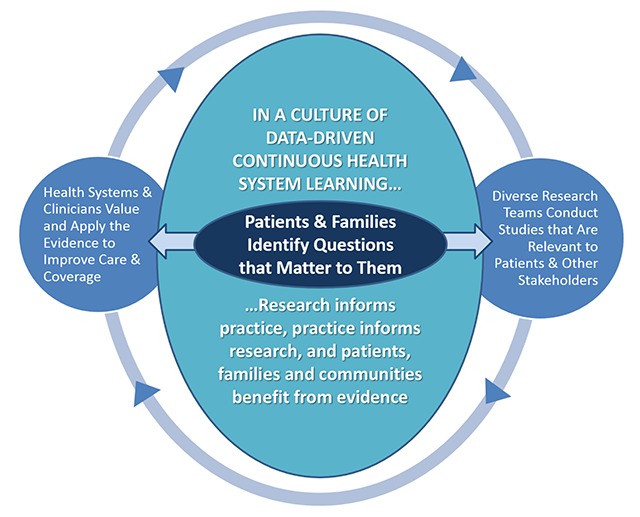
Data-Driven, Patient-Centered Learning Health System.

These steps are achievable if we build on our extant trusting relationships with delivery systems and communities, and share knowledge widely so that it can be applied and amplified. Only then can we overcome the inertia of translation and support patient-centered learning health systems (Figure 1).

## The next 25 years in health care research

Remarkably, the first publication about the HCSRN in 1998, (when it was still the HMO Research Network) opened with this statement: “Rapid and dramatic change in the health care industry has opened new doors and created expanded opportunities for health services research.” [19] It’s fair to say that this is an evergreen concept—advancements in technology and scientific discovery, with the competing need to control cost and improve quality, guarantee a continuous siege of changes in health care at the individual, population, and organizational level. In recent years, we’ve both witnessed, studied, and led changes in health care brought about by social media, wearables, genomic medicine, telemedicine, immunotherapy, augmented/virtual reality, and consolidation of health and hospital systems, while new “disrupters” aim to diversify options for patients. The potential for innovations is ongoing, but systematic and generalizable investigations into what works best for whom, under what circumstances are no less imperative than they were when medicine was still rudimentary. To the extent the entire research community can leverage a common infrastructure such as the HCSRN, populations will benefit and we’ll see a greater return on our investment in science.

## Appendix 1: HCSRN Special Issue Papers by Topic Area

### Introduction

- Greene, SM, et al. **We’ve Only Just Begun – Insights from a 25-Year Journey to Accelerate Health Care Transformation Through Delivery System Research**. eGEMs (Generating Evidence & Methods to improve patient outcomes), 2019; 7(1): 11. DOI: https://doi.org/10.5334/egems.310

### Effective Approaches to Collaboration

- Tuzzio, L, Larson, EB. **The Promise of Pragmatic Clinical Trials Embedded in Learning Health Systems**. eGEMs (Generating Evidence & Methods to improve patient outcomes). 2019; 7(1): 10. DOI: http://doi.org/10.5334/egems.285
- Doria-Rose, VP, Greenlee, RT, Buist, DSM, Miglioretti, DL, Corley, DA, Brown, JS, et al. **Collaborating on Data, Science, and Infrastructure: The 20-Year Journey of the Cancer Research Network**. eGEMs (Generating Evidence & Methods to improve patient outcomes). 2019; 7(1): 7. DOI: http://doi.org/10.5334/egems.273
- Rahm, AK, et al. **The Healthcare Systems Research Network (HCSRN) as an Environment for Dissemination and Implementation Research: A Case Study of Developing a Multi-Site Research Study in Precision Medicine**. eGEMs (Generating Evidence & Methods to improve patient outcomes). 2019; 7(1): 16, pp. 1–8. DOI: https://doi.org/10.5334/egems.283

### Optimizing Source Data from EHRs to Support Rigorous Research

- Benuzillo, J, Savitz, LA, Evans, S. **Improving Health Care with Advanced Analytics: Practical Considerations**. eGEMs (Generating Evidence & Methods to improve patient outcomes). 2019; 7(1): 3. DOI: http://doi.org/10.5334/egems.276
- Weeks, J, Pardee, R. **Learning to Share Health Care Data: A Brief Timeline of Influential Common Data Models and Distributed Health Data Networks in U.S. Health Care Research**. eGEMs (Generating Evidence & Methods to improve patient outcomes). 2019; 7(1): 4. DOI: http://doi.org/10.5334/egems.279
- Yu, O, Reed, SD, Schulze-Rath, R, Grafton, J, Hansen, K, Scholes, D. **Identification of Incident Uterine Fibroids Using Electronic Medical Record Data**. eGEMs (Generating Evidence & Methods to improve patient outcomes). 2019; 7(1): 5. DOI: http://doi.org/10.5334/egems.264
- Stewart, CC, et al. **Impact of ICD-10-CM Transition on Mental Health Diagnoses Recording**. eGEMs (Generating Evidence & Methods to improve patient outcomes). 2019; 7(1): 14, DOI: https://doi.org/10.5334/egems.281
- Sengupta, S, Bachman, D, Laws, R, Saylor, G, Staab, J, Vaughn, D, et al. **Data Quality Assessment and Multi-Organizational Reporting: Tools to Enhance Network Knowledge**. eGEMs (Generating Evidence & Methods to improve patient outcomes). 2019; 7(1): 8. DOI: http://doi.org/10.5334/egems.280
- Lin, PD, et al. **Comparing Prescribing and Dispensing Data of the PCORnet Common Data Model Within PCORnet Antibiotics and Childhood Growth Study**. eGEMs (Generating Evidence & Methods to improve patient outcomes). 2019; 7(1): 11. DOI: https://doi.org/10.5334/egems.274
- Simon, GE, Shortreed, SM, Coley, RY, Penfold, RB, Rossom, RC, Waitzfelder, BE, et al. **Assessing and Minimizing Re-identification Risk in Research Data Derived from Health Care Records**. eGEMs (Generating Evidence & Methods to improve patient outcomes). 2019; 7(1): 6. DOI: http://doi.org/10.5334/egems.270

### Applying Electronic Data for Point-of-Care Improvement

- Lieu, TA, Herrinton, LJ, Buzkov, DE, Liu, L, Lyons, D, Neugebauer, R, et al. **Developing a Prognostic Information System for Personalized Care in Real Time**. eGEMs (Generating Evidence & Methods to improve patient outcomes). 2019; 7(1): 2. DOI: http://doi.org/10.5334/egems.266
- Ekstrom, HL, et al. **Development of a Clinical Decision Support System for Pediatric Abdominal Pain in Emergency Department Settings Across Two Health Systems Within the HCSRN**. eGEMs (Generating Evidence & Methods to improve patient outcomes). 2019; 7(1): 15. DOI: https://doi.org/10.5334/egems.282
- Yarborough, BJH, et al. **Challenges of Population-based Measurement of Suicide Prevention Activities Across Multiple Health Systems**. eGEMs (Generating Evidence & Methods to improve patient outcomes). 2019; 7(1): 13. DOI: https://doi.org/10.5334/egems.277
- Sperl-Hillen, JM, Rossom, RC, Kharbanda, EO, Gold, R, Geissal, ED, Elliott, TE, et al. **Priorities Wizard: Multisite Web-Based Primary Care Clinical Decision Support Improved Chronic Care Outcomes with High Use Rates and High Clinician Satisfaction Rates**. eGEMs (Generating Evidence & Methods to improve patient outcomes). 2019; 7(1): 9. DOI: http://doi.org/10.5334/egems.284
- Bayliss, EA, et al. **Using Self-Reported Data to Segment Older Adult Populations with Complex Care Needs**. eGEMs (Generating Evidence & Methods to improve patient outcomes). 2019; 7(1): 12. DOI: https://doi.org/10.5334/egems.275

## References

[1] Kohn, KT, Corrigan, JM and Donaldson, MS. To Err Is Human: Building a Safer Health System Washington, DC: National Academy Press; 1999. 25077248

[2] Institute of Medicine (US) Committee on Quality of Health Care in America. Crossing the Quality Chasm: A New Health System for the 21st Century Washington (DC): National Academies Press (US); 2001. PubMed PMID: 25057539. 25057539

[3] Tricoci, P, Allen, JM, Kramer, JM, Califf, RM and Smith, SC, Jr. Scientific evidence underlying the ACC/AHA clinical practice guidelines. JAMA. 2009 2 25; 301(8): 831–41. Erratum In: *JAMA* 2009 Apr 15; 301(15): 1544. PubMed PMID: 19244190. DOI: 10.1001/jama.2009.205 19244190

[4] The clinical trials enterprise in the United States: a call for disruptive innovation In: Califf, RM, Filerman, GL, Murray, RK and Rosenblatt, M (eds.), Envisioning a transformed clinical trials enterprise in the United States: establishing an agenda for 2020 — workshop summary; 2012 Washington, DC: National Academies Press. 23236648

[5] Vogt, TM, Elston-Lafata, J, Tolsma, D and Greene, SM. The role of research in integrated healthcare systems: the HMO Research Network. Am J Manag Care. 2004 9; 10(9): 643–8. PubMed PMID: 15515997. 15515997

[6] Senge, PM. The Fifth Discipline: the Art and Practice of the Learning Organization New York: Doubleday/Currency; 1990.

[7] Laney, D. 3D Data Management: Controlling Data Volume, Velocity and Variety, Gartner Report; 2001 https://blogs.gartner.com/doug-laney/files/2012/01/ad949-3D-Data-Management-Controlling-Data-Volume-Velocity-and-Variety.pdf. Accessed January 2019.

[8] Hornbrook, MC, Hart, G, Ellis, JL, Bachman, DJ, Ansell, G, Greene, SM, Wagner, EH, Pardee, R, Schmidt, MM, Geiger, A, Butani, AL, Field, T, Fouayzi, H, Miroshnik, I, Liu, L, Diseker, R, Wells, K, Krajenta, R, Lamerato, L and Neslund Dudas, C. Building a virtual cancer research organization. J Natl Cancer Inst Monogr. 2005; 35: 12–25. PubMed PMID: 16287881. DOI: 10.1093/jncimonographs/lgi033 16287881

[9] Ross, TR, Ng, D, Brown, JS, Pardee, R, Hornbrook, MC, Hart, G and Steiner, JF. The HMO Research Network Virtual Data Warehouse: A Public Data Model to Support Collaboration EGEMS (Wash DC). 2014 3 24; 2(1): 1049 eCollection 2014. PubMed PMID: 25848584; PubMed Central PMCID: PMC4371424. DOI: 10.13063/2327-9214.1049 25848584 PMC4371424

[10] Zapka, JG, Taplin, SH, Solberg, LI and Manos, MM. A framework for improving the quality of cancer care: the case of breast and cervical cancer screening. Cancer Epidemiol Biomarkers Prev. 2003 1; 12(1): 4–13. Review. PubMed PMID: 12540497. 12540497

[11] Vogel, AL, Hall, KL, Fiore, SM, Klein, JT, Bennett, LM, Gadlin, H, Stokols, D, Nebeling, LC, Wuchty, S, Patrick, K, Spotts, EL, Pohl, C, Riley, WT and Falk-Krzesinski, HJ. The Team Science Toolkit: enhancing research collaboration through online knowledge sharing. Am J Prev Med. 2013 12; 45(6): 787–9. PubMed PMID: 24237924. DOI: 10.1016/j.amepre.2013.09.001 24237924

[12] Institute of Medicine (US) Roundtable on Value and Science-Driven Health Care. Learning What Works: Infrastructure Required for Comparative Effectiveness Research: Workshop Summary Washington: National Academies Press (US); 2011. PubMed PMID: 22013609. 22013609

[13] Greene, SM, Reid, RJ and Larson, EB. Implementing the learning health system: from concept to action. Ann Intern Med. 2012 8 7; 157(3): 207–10. PubMed PMID: 22868839. DOI: 10.7326/0003-4819-157-3-201208070-00012 22868839

[14] Forrest, CB, Chesley, FD, Jr., Tregear, ML and Mistry, KB. Development of the Learning Health System Researcher Core Competencies. Health Serv Res. 2017 8 4 [Epub ahead of print] PubMed PMID: 28777456; PubMed Central PMCID: PMC6051975. DOI: 10.1111/1475-6773.12751 PMC6051975 28777456

[15] Arterburn, D, Wellman, R, Emiliano, A, Smith, SR, Odegaard, AO, Murali, S, Williams, N, Coleman, KJ, Courcoulas, A, Coley, RY, Anau, J, Pardee, R, Toh, S, Janning, C, Cook, A, Sturtevant, J, Horgan, C and McTigue, KM. PCORnet Bariatric Study Collaborative. Comparative Effectiveness and Safety of Bariatric Procedures for Weight Loss: A PCORnet Cohort Study. Ann Intern Med. 2018 12 4; 169(11): 741–750. Epub 2018 Oct 30. PubMed PMID: 30383139. DOI: 10.7326/M17-2786 30383139 PMC6652193

[16] Demakis, JG, McQueen, L, Kizer, KW and Feussner, JR. Quality Enhancement Research Initiative (QUERI): A collaboration between research and clinical practice. Med Care. 2000 6; 38(6 Suppl 1): I17–25. Review. PubMed PMID: 10843267. 10843267

[17] Atkins, D, Kilbourne, AM and Shulkin, D. Moving From Discovery to System-Wide Change: The Role of Research in a Learning Health Care System: Experience from Three Decades of Health Systems Research in the Veterans Health Administration. Annu Rev Public Health. 2017 3 20; 38: 467–487. Epub 2017 Jan 11. Review. PubMed PMID: 28125386. DOI: 10.1146/annurev-publhealth-031816-044255 28125386

[18] Kilbourne, AM, Elwy, AR, Sales, AE and Atkins, D. Accelerating Research Impact in a Learning Health Care System: VA’s Quality Enhancement Research Initiative in the Choice Act Era. Med Care. 2017 7; 55(Suppl 7 Suppl 1): S4–S12. PubMed PMID: 27997456; PubMed Central PMCID:PMC5472006. DOI: 10.1097/MLR.0000000000000683 PMC5472006 27997456

[19] Durham, ML. Partnerships for research among managed care organizations. Health Aff (Millwood). 1998 Jan-Feb; 17(1): 111–22. PubMed PMID: 9455021. DOI: 10.1377/hlthaff.17.1.111 9455021

